# Protective Effect of Red Light-Emitting Diode against UV-B Radiation-Induced Skin Damage in SKH:HR-2 Hairless Mice

**DOI:** 10.3390/cimb46060338

**Published:** 2024-06-06

**Authors:** Eun-Chae Cho, Surin Ahn, Kyung-Ok Shin, Joon Byeong Lee, Hyo-Jeong Hwang, Yean-Jung Choi

**Affiliations:** 1 Department of Convergence Science, Sahmyook University, Seoul 01795, Republic of Korea; ec.cho0201@gmail.com (E.-C.C.); surin1202@naver.com (S.A.); 2 Department of Food and Nutrition, Sahmyook University, Seoul 01795, Republic of Korea; skorose@syu.ac.kr; 3 Aventech Inc., Suwon 16522, Gyeonggi-do, Republic of Korea

**Keywords:** red light-emitting diode, ultraviolet, skin damage, collagen, elastin

## Abstract

In this in vivo study on hairless mice, we examined the effects of light-emitting diode (LED) treatment applied prior to ultraviolet B (UVB) irradiation. We found that pre-treating with LED improved skin morphological and histopathological conditions compared to those only exposed to UVB irradiation. In our study, histological evaluation of collagen and elastic fibers after LED treatment prior to UVB irradiation showed that this pretreatment significantly enhanced the quality of fibers, which were otherwise poor in density and irregularly arranged due to UV exposure alone. This suggests that LED treatment promotes collagen and elastin production, leading to improved skin properties. Additionally, we observed an increase in Claudin-1 expression and a reduction in nuclear factor-erythroid 2-related factor 2 (Nrf-2) and heme-oxygenase 1 (HO-1) expression within the LED-treated skin tissues, suggesting that LED therapy may modulate key skin barrier proteins and oxidative stress markers. These results demonstrate that pretreatment with LED light can enhance the skin’s resistance to UVB-induced damage by modulating gene regulation associated with skin protection. Further investigations are needed to explore the broader biological effects of LED therapy on other tissues such as blood vessels. This study underscores the potential of LED therapy as a non-invasive approach to enhance skin repair and counteract the effects of photoaging caused by UV exposure.

## 1. Introduction

The skin serves as the primary barrier against harmful external environmental factors, including pathogens, chemicals, air pollutants, and notably, solar ultraviolet (UV) radiation. This outermost layer of the human body is integral to defending against the various agents that contribute to aging [1,2]. Skin aging can be categorized into two types: intrinsic aging, which is primarily governed by genetic factors and occurs naturally over time, and extrinsic aging, predominantly caused by environmental influences such as UVB radiation [3,4].

Prolonged and excessive exposure to UV light not only triggers acute conditions like burns and immunosuppression but also leads to persistent skin damage, potentially resulting in skin cancer and photoaging [5,6]. The process of photoaging is characterized by symptoms such as wrinkles, loss of elasticity, pigmentation, brown spots, and skin thickening [7,8]. These changes are primarily driven by the activation of skin cells such as keratinocytes and fibroblasts under UV exposure, which produce reactive oxygen species (ROS) [9,10]. These ROS stimulate intracellular signaling pathways that disrupt the balance of collagen and elastic fibers in the skin—collagen fibers collapse, while there is an abnormal accumulation of substances containing elastin [11]. Biochemical connective tissue changes in the dermis are influenced by the differing responses of collagen types I and III to UV radiation [12]. While both types are crucial structural components, type III collagen, less dense and simpler in structure, exhibits greater susceptibility to UV-induced degradation compared to the denser, more cross-linked type I collagen. This differential susceptibility can be attributed to the unique structural and biochemical properties of each collagen type, alongside their regulation by matrix metalloproteinases (MMPs), which are upregulated in response to UV exposure [13,14]. Pro-collagen molecules, common to both collagen types, undergo a decrease in type I and an increase in type III within the dermis, driven by environmental factors such as UV radiation [15], suggesting different roles and molecular stabilities that warrant further investigation into photoaging mechanisms and therapeutic strategies.

In our investigation, particular attention was given to key proteins such as Claudin-1, nuclear factor erythroid 2-related factor 2 (Nrf-2), and heme oxygenase-1 (HO-1), which are pivotal in maintaining skin integrity and function. Claudin-1 is a critical component of tight junctions in the skin, playing a vital role in barrier function and hydration by preventing transepidermal water loss and shielding against external pathogens and chemicals [16]. Meanwhile, Nrf-2 and HO-1 are integral to the skin’s oxidative stress response [17]. Under UV exposure, oxidative stress can lead to the breakdown of structural proteins such as collagen and elastin, crucial for skin elasticity and firmness. Nrf-2 acts as a master regulator of antioxidant response, modulating the expression of protective genes including HO-1, which provides cytoprotection against oxidative damage. Together, these proteins support the structural integrity and resilience of the skin against environmental stressors, such as UV radiation, that precipitate aging [18]. Thus, understanding their roles and interactions provides essential insight into the mechanisms of skin aging and the potential of therapies aimed at mitigating photoaging effects. This background underscores the relevance of our study, which explores the impact of LED treatment on these proteins within the context of UVB-irradiated skin, aiming to harness their regenerative and protective capabilities to counteract photoaging.

Previous studies have highlighted a significant gap in data concerning how these molecular and biochemical processes linked to UV exposure contribute to the broader context of skin function and aging [19]. This study aims to bridge this gap by exploring the efficacy of near-infrared light-emitting diode (LED) technology, specifically a 630 nm wavelength, in penetrating the dermal layer and improving these photoaging symptoms. LEDs are semiconductor light-emitting devices that have recently become widely used [13]. Infrared LEDs, in particular, are used in many applications, including agricultural product dryers, muscle treatment devices, air purifiers, LED masks and brushes, and notably in medical treatments such as photodynamic therapy for skin [20,21,22]. Using hairless mice repeatedly exposed to UV light, we investigate whether LED treatment can mitigate the effects of UV-induced aging by promoting skin regeneration and altering molecular pathways associated with aging.

In summary, this research evaluated the anti-photoaging properties of LED treatment by specifically analyzing its effects on UVB-induced photoaging. We aimed to delineate the molecular mechanisms involved by focusing on key proteins such as Claudin-1, Nrf-2, and HO-1, which play crucial roles in the skin’s defense against environmental stressors like UV radiation. By measuring these proteins in UVB-irradiated Skh:HR-1 hairless mice, we seek to understand how LED technology can potentially reverse photoaging symptoms and offer new directions for clinical and therapeutic anti-aging interventions.

## 2. Materials and Methods

### 2.1. Ethical Statement and Animals

All animal experiments were performed according to the protocols approved by the Animal Care and Use Committee of Sahmyook University (SYUIACUC 2022-004). Male, 5-week-old Skh:HR-1 hairless mice were obtained from DooYeol Biotech Co., Ltd. (Seoul, Republic of Korea). Mice were bred in a controlled standard environment (22 ± 2 °C, 50 ± 10% relative humidity, 12 h light/dark cycle) in the animal research facility of Sahmyook University. The mice were fed a commercial unrefined rodent diet and tap water ad libitum.

### 2.2. Experiment Design and Treatment

**Figure 1:**
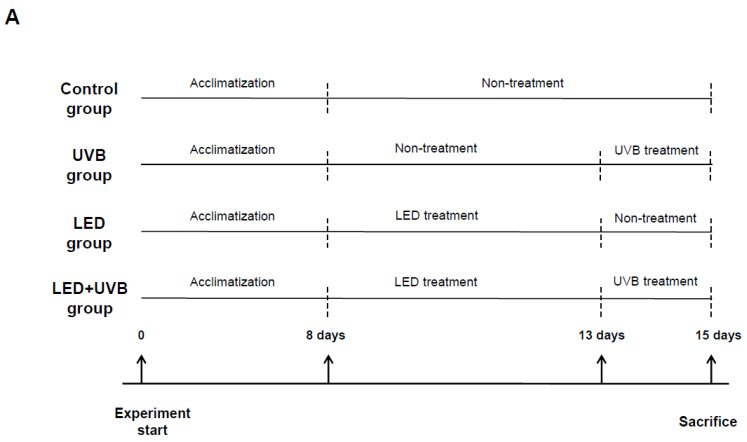

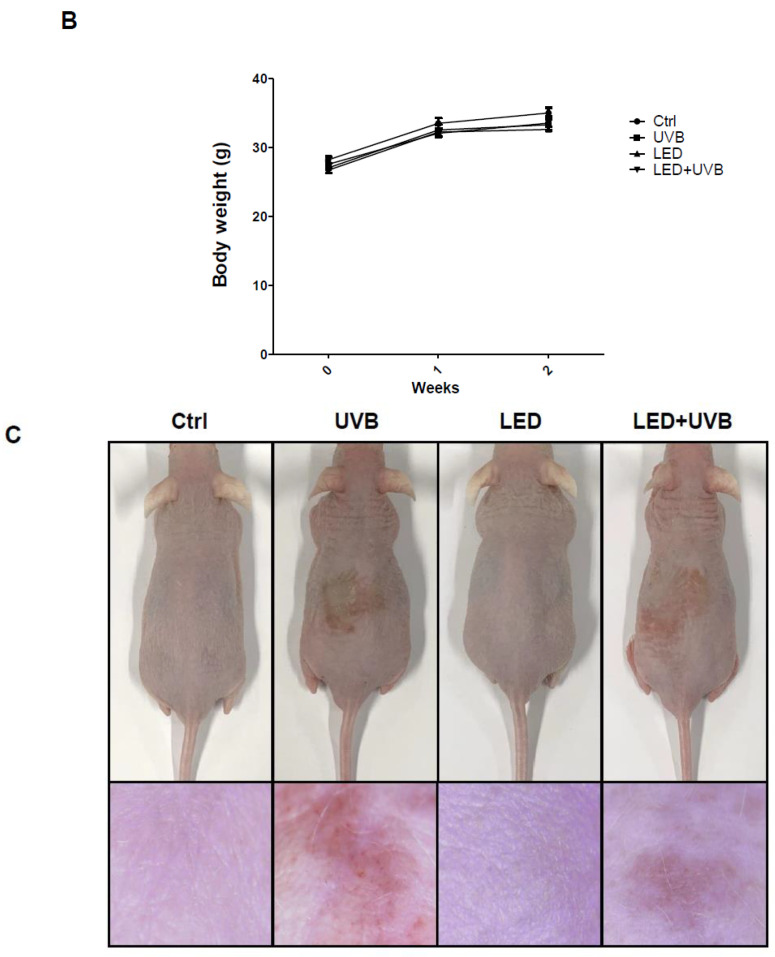
(**A**) Animal experiment design and (**B**) body weight changes in hairless mice treated with test materials. (**C**) The mice were exposed to light-emitting diode (LED) light (630 nm) for 30 min twice daily for five days. All mice, except those in the control group, were irradiated with 200 mJ/cm^2^ UVB once a day for three days before sacrifice. At the end of the experimental period, dorsal skin tissues were immediately collected for further analysis. Each value represents the mean ± SD (n = 5).

After acclimatization for one week, Skh:HR-1 hairless mice were randomly divided into four groups as follows: (i) control group (no UVB or LED irradiation, designated Ctrl), (ii) UVB irradiation group (designated UV), (iii) LED irradiation group (designated LED), and (iv) combined UVB and LED irradiation group (designated UV + LED) (Figure 1A). We used an LED brush (FDS-CB001, 68 L × 113 W × 61.5 H) supplied by Higgs Korea Co., Ltd. (Anyang, Republic of Korea), administering LED irradiation at 630 nm for 30 min twice daily for five days. Mice were anesthetized using a respiratory anesthetic (Hana Pharm Co., Ltd., Seoul, Republic of Korea) and subjected to UV irradiation lamps (VL-6MC, Vilber Lourmat, Collégien, France) emitting 312 nm UVB. The UVB dosage was established according to previous studies [23,24], set at 200 mJ/cm^2^ (Figure 1B,C). All mice, except those in the control group, were irradiated with UVB once daily for a total of three days before sacrifice. At the end of the experimental period, dorsal skin tissues were immediately collected for further analysis.

### 2.3. Hematoxylin and Eosin (H&E), Masson’s Trichrome, and Elastic Connective Tissue Staining

Histopathological observations of the dorsal skin were performed using an H&E staining kit, trichrome stain kit (connective tissue stain), and elastic stain kit (Verhoeff Van Gieson [EVG] stain). All staining was performed according to the manufacturer’s instructions (Abcam).

### 2.4. Western Blot Analysis

The total dorsal skin proteins were dissolved to assess the changes in various skin factor expressions induced by UVB and LED irradiation. Dorsal skin tissue was dissolved as previously described [25]. The protein content of skin tissue lysates was determined using a bicinchoninic acid (BCA) protein assay kit (Thermo Fisher Scientific Inc., Waltham, MA, USA). Western blotting was performed as previously described [26]. Equal amounts of protein were separated by sodium dodecyl-sulfate polyacrylamide gel electrophoresis (SDS-PAGE). Separated proteins were then transferred onto polyvinylidene fluoride (PVDF) membranes by electroblotting. Primary and secondary antibodies were added to the PVDF membrane and the changes in the expression of specific proteins were analyzed using Immobilon Western chemiluminescent horseradish peroxidase (HRP) substrate (Millipore Corp., Billerica, MA, USA). Claudin-1 antibody was purchased from Abcam (Waltham, MA, USA), antibodies against nuclear factor erythroid 2-related factor 2 (Nrf-2) and heme oxygenase-1 (HO-1) antibodies were purchased from Cell Signaling Technology (Beverly, MA, USA), and β-actin antibody was purchased from Santa Cruz Biotechnology, Inc. (Dallas, TX, USA). The relative expression of the target proteins was estimated using a KwikQuant Imager (Kindle Biosciences LLC, CT, USA) and normalized to β-actin.

### 2.5. Statistical Analysis

All data obtained from the study are expressed as mean ± standard deviation (SD) from at least three independent experiments. To assess the normality of data distribution, the Shapiro–Wilk test was performed prior to further statistical analyses. This preliminary step ensured that the application of parametric tests was appropriate for our dataset. Subsequent analyses involved one-way and two-way analysis of variance (ANOVA), which were utilized to compare mean differences across multiple groups. Where significant differences were indicated, Bonferroni’s multiple comparison test was conducted to identify specific group contrasts. Statistical significance was established at a *p*-value of <0.05.

## 3. Results

### 3.1. Histopathological Changes by LED Treatment in UVB-Irradiated Hairless Mice

**Figure 2:**
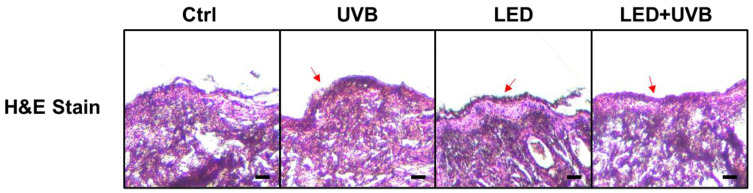
Effect of light emitting diode (LED) treatment on histological changes in ultraviolet (UVB)-irradiated skin of Skh:HR-1 hairless mice. Hematoxylin and eosin (H&E) staining of dorsal skin in the control, UV, LED, and UV + LED groups. The mice were exposed to LED light (630 nm) for 30 min twice daily for five days. All mice, except those in the control group, were irradiated with 200 mJ/cm^2^ UVB once a day for three days before sacrifice. Dorsal skin tissues were excised, and paraffin sections were prepared. Changes in skin tissue were measured using a corresponding staining kit. H&E staining revealed that the group treated with LED + UVB exhibited reduced epidermal thickening and fewer inflammatory cells compared to the UVB-only group, indicating that both epidermal and dermal damage were alleviated (red arrows). Scale bars, 100 μm.

Tissue staining was used to evaluate histopathological changes in each test group. H&E staining revealed that the epidermis of the control group was smooth and the tissue arrangement constituting the dermis layer was regular, whereas in the UVB group, the epidermal layer was thicker than the control group and the tissue arrangement in the dermis layer was irregular along with inflammation-related cells (Figure 2). In the UVB + LED group, epidermal thickening and inflammatory cells were reduced compared with those in the UVB group, and epidermal and dermal damage was alleviated, showing a pattern similar to that of the control group.

**Figure 3:**
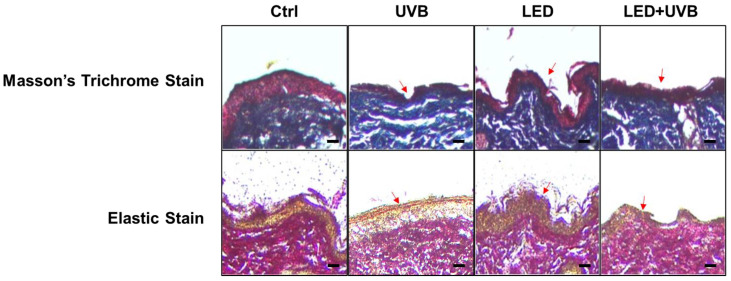
Effect of light emitting diode (LED) treatment on collagen fibers and elastin fibers in ultraviolet (UV)B-irradiated skin of Skh:HR-1 hairless mice. Masson’s trichrome staining of the dorsal skin reveals collagen deposition (blue). Elastic staining of the dorsal skin revealed elastin fibers (blue-black). The mice were exposed to LED light (630 nm) for 30 min twice daily for five days. All mice, except those in the control group, were irradiated with 200 mJ/cm^2^ UVB once a day for three days before sacrifice. Dorsal skin tissues were excised, and paraffin sections were prepared. Changes in skin tissue were measured using a relevant staining kit. Masson’s trichrome staining demonstrated that in the LED + UVB treated group, regeneration of damaged dermal tissue was evident, with marked formation of collagen fibers and improved dermal tissue morphology (red arrows). Elastic staining indicated that the elastin content in the LED + UVB treated group was comparable to that of the control group, as highlighted by red arrows. Scale bars, 100 μm.

Masson’s trichrome staining confirmed the change in collagen composition in the dermal layer (Figure 3). Collagen fibers were regularly formed in the control group; however, in the UVB group, collagen fibers were destroyed by UV irradiation and exhibited an irregular arrangement. In the UVB + LED group, the damaged dermal tissue regeneration was effectively reflected by evident collagen fiber tissue formation and dermal tissue morphology. Finally, elastic staining (Figure 3), revealed a loss of elastin in tissues in the UVB group compared to the control group, whereas the UVB + LED group exhibited a similar degree of elastin content to the control group.

### 3.2. Changes in Claudin-1 Expression on Dorsal Skin by LED Treatment in UVB-Irradiated Hairless Mice

**Figure 4:**
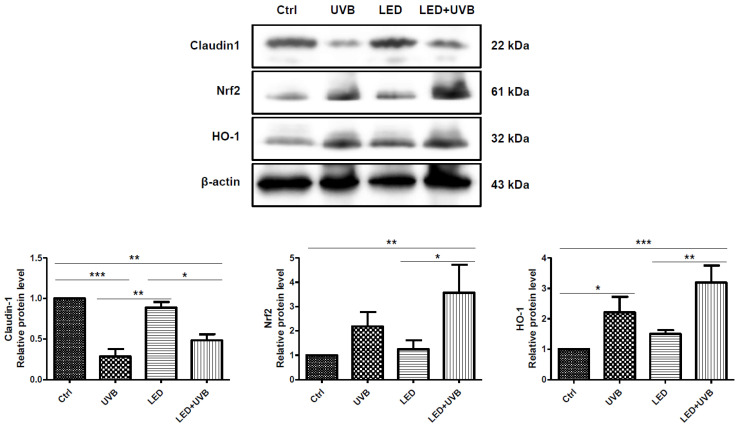
Effect of light-emitting diode (LED) treatment on Claudin-1 and Nrf2/HO-1 signaling pathway in ultraviolet (UV)B-irradiated skin of Skh:HR-1 hairless mice. The mice were exposed to LED light (630 nm) for 30 min twice daily for five days. All mice, except those in the control group, were irradiated with 200 mJ/cm^2^ UVB once a day for three days before sacrifice. Dorsal skin tissues were excised and homogenized. Total lysates of dorsal skin tissues were prepared and analyzed by Western blot using the indicated antibodies. The Western blot images are representative of three independent experiments. Quantitative analysis of the Western blot results. Protein expression was normalized to β that of-actin and presented relative to the control group. Each value represents the mean ± SD (n = 5). * *p* < 0.05, ** *p* < 0.01, *** *p* < 0.001 significantly different from the control group.

Western blotting was used to confirm whether there was a skin barrier strengthening function, or an antioxidant-related factor stimulation function inferred by LED treatment. The skin barrier protection mechanism caused by LED treatment was confirmed by the quantitative change in claudin-1, a component of tight junction proteins (Figure 4). As a result, it was confirmed that the claudin-1 protein expression level was significantly decreased in the UVB group compared to the control group (*p* < 0.001). Although the increase in the expression level was not significant in the UVB + LED group compared to that in the UVB group, an increase was observed compared to that in the UVB group.

### 3.3. Changes in Nrf-2 and HO-1 Expression in the Dorsal Skin by LED Treatment in UVB-Irradiated Hairless Mice

In this study, we investigated whether LED treatment is involved in the quantitative changes in Nrf2 and HO-1 proteins affected by oxidative stress caused by UVB irradiation (Figure 4). Although not statistically significant, the Nrf2 protein expression level increased in the UVB group compared to that in the control group. In the UVB + LED group, the Nrf2 protein expression level increased more than that in the UVB group, which was a significant increase compared to the control group (*p* < 0.01) and the LED group (*p* < 0.05), but not compared to the UVB group.

HO-1 protein expression was significantly higher in the UVB group than in the control group (*p* < 0.05). HO-1 protein expression also increased more in the UVB + LED group than in the UVB group, showing a significant increase compared to the control group (*p* < 0.001) and the LED group (*p* < 0.01) but not compared to the UVB group.

## 4. Discussion

We present four main findings obtained from this study: (1) In the pathological examination of skin tissues irradiated with 200 mJ/cm^2^ UVB, LED treatment was found to prevent skin thickness and dermal layer damage, which are typical symptoms of skin photoaging. (2) Following LED treatment, low-density and irregularly arranged collagen and elastic fibers improved to a level similar to that of the control group. (3) LED treatment prevented skin barrier damage by increasing Claudin-1 protein expression in UVB-irradiated skin tissues. (4) LED treatment suggests that the main cause of skin damage due to oxidative stress during photoaging is ameliorated by upregulating the Nrf2/HO-1 pathway. Therefore, LED treatment may be effective in reversing UVB-induced photoaging by attenuating photoaging-related histopathological changes and regulating collagen/elastin replacement, skin barrier, and antioxidant-related pathways.

In response to the growing interest in anti-aging skincare, driven by an aging population and increased economic capability [27], our study delves into the effects of LED therapy on photoaging—a significant aspect of skin aging predominantly caused by UV rays (UVA and UVB). Photoaging typically manifests more severely and prematurely than chronological aging, highlighted by changes such as increased skin laxity and the formation of wrinkles [28,29,30]. Our research specifically explored the use of infrared LED therapy, a modern technique increasingly adopted in home care settings, to mitigate the effects of photoaging [31,32]. We employed a 630 nm wavelength LED to examine its impact on UVB-induced skin damage. Pathological examination of skin tissues pretreated with LED prior to UVB irradiation demonstrated that the LED treatment effectively prevented the increase in skin thickness and mitigated damage to the dermal layer. This preventive action contributed to the maintenance of skin properties, reducing the severity of photoaging symptoms and promoting skin health. This consistency with prior research supports the efficacy of LED therapy in clinical applications for skin rejuvenation [33], offering a promising non-invasive solution for reducing the signs of aging induced by UV exposure. In our discussion, it is pertinent to consider recent findings from other studies that echo and contextualize our results regarding LED therapy for skin rejuvenation. Recent studies on LED phototherapy for skin aging have underscored the effectiveness of this treatment in combating UV-induced damage. These studies explored both the protective and therapeutic potential of LED therapy, particularly focusing on its benefits in addressing photoaging. Huang et al. [33] highlighted the broad applicability and safety of LED phototherapy in treating aging skin, focusing on its minimally invasive nature and potential for combination with other treatments. Their emphasis on the intrinsic cellular activities modified by LED therapy aligns with our observations of improved skin morphologies and repair mechanisms post-treatment. Couturaud et al. [13] provided compelling evidence of red light photobiomodulation’s effectiveness over a 3-month period, showcasing significant anti-aging effects such as reduced wrinkle depth and improved skin elasticity, measured through various sophisticated techniques. These results not only support but enhance our understanding of the duration and progressive nature of LED therapy benefits, which we observed in the prevention of UVB-induced skin damage. Sorbellini et al. [34] reviewed the broad application of LED therapy in dermatology, affirming its safety and efficacy for various skin conditions, including aging. This review supports the foundational role of LED in dermatological treatments and underscores the need for continued research, particularly in controlled settings, to better establish treatment parameters and outcomes. Together, these studies reinforce our findings that pretreatment with LED can effectively shield skin from UVB-induced damage, while also promoting rejuvenation and repair. The consistent improvements in skin quality across different studies suggest that LED therapy, particularly when applied before harmful exposure, contributes significantly to dermatological health, supporting its integration into preventative skin care regimens. These outcomes underscore the potential of LED therapy as a valuable addition to the repertoire of anti-aging skincare treatments.

Exposure to UV rays significantly increases the production of reactive oxygen species (ROS) in the skin, which in turn causes damage to nucleic acids and proteins, and promotes gene mutations [35]. This oxidative stress escalates the activity of matrix metalloproteinases (MMPs), enzymes that degrade collagen and elastin—the fibrous proteins responsible for the skin’s structural integrity and elasticity. Consequently, the degradation of these proteins leads to the formation of wrinkles and other signs of skin aging [36]. Elastin, though less abundant than collagen, plays a critical role in skin elasticity by forming a supportive network within the epidermis [37,38,39,40]. The production of elastase, which breaks down elastin, ceases around the age of 18–19 years, making any subsequent loss of elastin difficult to recover [41,42]. In our study, LED treatment applied prior to UVB exposure effectively preserved the density and arrangement of collagen and elastic fibers, maintaining them at levels similar to those observed in the control group. This not only ameliorated photoaging symptoms but also facilitated skin regeneration. These findings align with previous research, which has also highlighted the potential of LED therapy in reversing the structural damage caused by UV exposure and promoting the recovery of skin health [43].

In this study, we investigated the protective effects of LED treatment on the skin barrier, particularly through the modulation of Claudin-1 expression in UVB-irradiated skin tissues. The skin barrier, primarily composed of the stratum corneum with layers of dead keratinocytes (corneocytes) and intercellular lipids, serves as a critical shield against external environmental stressors and prevents moisture loss, crucial for maintaining overall skin health [44,45]. Damage to this barrier, often exacerbated by aging or external factors such as UV radiation, can lead to increased skin dehydration and the formation of wrinkles [46,47]. Tight junctions, which include components like occludin, claudin, junctional adhesion molecules (JAMs), and zona occludens (ZOs), are essential for maintaining the integrity and function of the skin barrier [48,49]. They facilitate cell-to-cell connections, regulate the passage of substances between cells, and help maintain cellular polarity. Our findings reveal that LED treatment notably enhances the expression of Claudin-1, a key protein in tight junctions, thereby strengthening the skin barrier’s resistance to UVB-induced damage. These results are consistent with prior studies that have highlighted the effectiveness of red LED light in improving skin barrier functions and mitigating damage caused by environmental factors such as UV radiation [50,51]. By increasing Claudin-1 expression, LED therapy not only helps in preserving the structural and functional integrity of the skin barrier but also suggests a viable therapeutic approach for enhancing skin health and combating photoaging. This study underscores the potential of LED treatment as a non-invasive method to protect and regenerate the skin by modulating critical proteins involved in barrier function.

In this study, we explored how LED treatment influences the Nrf2/HO-1 signaling pathway, which plays a crucial role in mitigating oxidative stress-induced skin damage, a common component of photoaging. Reactive oxygen species (ROS) are byproducts of cellular metabolism in living organisms. While low levels of ROS are beneficial for cellular functions such as proliferation, excessive ROS can lead to cell damage, death [52,53,54], and contribute to diseases like cancer, hypertension, and Alzheimer’s disease [55,56], underscoring the importance of regulatory mechanisms like the Nrf2/HO-1 pathway. Nrf2 is a regulator that protects against oxidative stress by upregulating antioxidant response elements (AREs) in the presence of oxidative stress, thus enhancing the body’s defense system. HO-1, an enzyme regulated by Nrf2, offers protection against oxidative stress by exerting antioxidant, anti-inflammatory, and anti-apoptotic effects [55,57,58]. In our findings, LED treatment appeared to activate this pathway, enhancing the skin’s resilience against oxidative damage typically exacerbated by UV exposure. This activation involves the translocation of Nrf2 from the cytosol to the nucleus, where it binds to AREs and promotes the expression of genes that bolster the skin’s antioxidant defenses, such as HO-1 [59,60]. These results are corroborated by previous research, confirming that enhancing the Nrf2/HO-1 pathway can significantly mitigate oxidative stress impacts in skin cells, helping to prevent the degradation of crucial structural components like collagen and elastin [61,62]. This suggests that LED therapy may be a viable non-invasive strategy to improve skin health and combat photoaging by leveraging the body’s natural defense mechanisms against oxidative stress. This parallels findings in other studies [63,64,65], underscoring the potential of Nrf2/HO-1 upregulation as a therapeutic target for protecting against environmental stressors in dermatological applications.

In this study, UVB irradiation was found to activate the Nrf2/HO-1 pathway, which plays a crucial role in mitigating oxidative stress-induced damage in skin cells. The application of LED treatment further enhanced the activity of this pathway, underscoring its potential to significantly bolster the skin’s defenses against oxidative stress. This suggests that red LED light treatment not only enhances the skin’s protective mechanisms but may also reduce the oxidative damage caused by UVB exposure through the upregulation of Nrf2/HO-1 activity. However, while the benefits of stimulating the Nrf2/HO-1 pathway are clear, it is essential to consider the balance of Nrf2 activity. Over-activation of the Nrf2 pathway has been linked in some studies to increased proliferation of cancer cells and resistance to chemotherapy. Therefore, additional research is needed to explore whether red LED light can directly inhibit ROS production without excessively activating Nrf2. Such studies will help clarify the optimal parameters for LED therapy that maximize therapeutic benefits while minimizing potential risks. This dual approach will ensure that LED treatment can be safely integrated into broader dermatological applications, particularly for conditions exacerbated by oxidative stress.

## 5. Conclusions

In this study, we applied a 630 nm near-infrared LED brush to hairless mice subjected to UVB irradiation to assess its efficacy in mitigating photoaging. Our findings confirm that LED treatment significantly restored skin thickness and repaired damage in the dermal layer, demonstrating its effectiveness in reversing the histopathological changes associated with photoaging. Notably, the treatment improved the density and arrangement of collagen and elastic fibers to levels comparable to those of the control group, enhancing the structural integrity of the skin. Furthermore, LED treatment bolstered the skin barrier by increasing Claudin-1 protein expression and ameliorated oxidative stress-induced skin damage through the upregulation of the Nrf2/HO-1 pathway. These results underscore the potential of LED treatment as a functional therapeutic approach for combating UV-induced skin damage, offering promising implications for clinical applications in skin care.

## Data Availability

The data used to support of this study are included with the article.

## References

[1] Harris-Tryon T.A., Grice E.A. (2022). Microbiota and maintenance of skin barrier function. Science.

[2] Sarandy M.M., Gonçalves R.V., Valacchi G. (2024). Cutaneous Redox Senescence. Biomedicines.

[3] Krutmann J., Schikowski T., Morita A., Berneburg M. (2021). Environmentally-Induced (Extrinsic) Skin Aging: Exposomal Factors and Underlying Mechanisms. J. Investig. Dermatol..

[4] Franco A.C., Aveleira C., Cavadas C. (2022). Skin senescence: Mechanisms and impact on whole-body aging. Trends Mol. Med..

[5] Leiter U., Keim U., Garbe C. (2020). Epidemiology of Skin Cancer: Update 2019. Adv. Exp. Med. Biol..

[6] Nanz L., Keim U., Katalinic A., Meyer T., Garbe C., Leiter U. (2024). Epidemiology of Keratinocyte Skin Cancer with a Focus on Cutaneous Squamous Cell Carcinoma. Cancers.

[7] Chen X., Yang C., Jiang G. (2021). Research progress on skin photoaging and oxidative stress. Postep. Dermatol. Alergol..

[8] Chen S., He Z., Xu J. (2020). Application of adipose-derived stem cells in photoaging: Basic science and literature review. Stem Cell Res. Ther..

[9] Gruber F., Kremslehner C., Eckhart L., Tschachler E. (2020). Cell aging and cellular senescence in skin aging—Recent advances in fibroblast and keratinocyte biology. Exp. Gerontol..

[10] Roh E., Kim J.E., Kwon J.Y., Park J.S., Bode A.M., Dong Z., Lee K.W. (2017). Molecular mechanisms of green tea polyphenols with protective effects against skin photoaging. Crit. Rev. Food Sci. Nutr..

[11] Pedić L., Pondeljak N., Šitum M. (2020). Recent information on photoaging mechanisms and the preventive role of topical sunscreen products. Acta Dermatovenerol. Alp. Pannonica Adriat..

[12] Pondeljak N., Lugović-Mihić L., Tomić L., Parać E., Pedić L., Lazić-Mosler E. (2023). Key Factors in the Complex and Coordinated Network of Skin Keratinization: Their Significance and Involvement in Common Skin Conditions. Int. J. Mol. Sci..

[13] Couturaud V., Le Fur M., Pelletier M., Granotier F. (2023). Reverse skin aging signs by red light photobiomodulation. Skin Res. Technol..

[14] Aziz J., Shezali H., Radzi Z., Yahya N.A., Abu Kassim N.H., Czernuszka J., Rahman M.T. (2016). Molecular Mechanisms of Stress-Responsive Changes in Collagen and Elastin Networks in Skin. Skin Pharmacol. Physiol..

[15] Tanveer M.A., Rashid H., Tasduq S.A. (2023). Molecular basis of skin photoaging and therapeutic interventions by plant-derived natural product ingredients: A comprehensive review. Heliyon.

[16] Bäsler K., Bergmann S., Heisig M., Naegel A., Zorn-Kruppa M., Brandner J.M. (2016). The role of tight junctions in skin barrier function and dermal absorption. J. Control. Release.

[17] Han S.H., Ballinger E., Choung S.Y., Kwon J.Y. (2022). Anti-Photoaging Effect of Hydrolysates from Pacific Whiting Skin via MAPK/AP-1, NF-κB, TGF-β/Smad, and Nrf-2/HO-1 Signaling Pathway in UVB-Induced Human Dermal Fibroblasts. Mar. Drugs..

[18] Oh S., Zheng S., Fang M., Kim M., Bellere A.D., Jeong J., Yi T.H. (2023). Anti-Photoaging Effect of Phaseolus angularis L. Extract on UVB-Exposed HaCaT Keratinocytes and Possibilities as Cosmetic Materials. Molecules.

[19] Salminen A., Kaarniranta K., Kauppinen A. (2022). Photoaging: UV radiation-induced inflammation and immunosuppression accelerate the aging process in the skin. Inflamm. Res..

[20] Glass G.E. (2021). Photobiomodulation: A review of the molecular evidence for low level light therapy. J. Plast. Reconstr. Aesthet. Surg..

[21] Rocha Mota L., Motta L.J., Duarte I.D.S., Horliana A.C.R.T., Silva D.F.T.D., Pavani C. (2018). Efficacy of phototherapy to treat facial ageing when using a red versus an amber LED: A protocol for a randomised controlled trial. BMJ Open.

[22] Cohen M., Austin E., Masub N., Kurtti A., George C., Jagdeo J. (2022). Home-based devices in dermatology: A systematic review of safety and efficacy. Arch. Dermatol. Res..

[23] Lim H.S., Yoon K., Lee D.H., Lee Y.S., Chung J.H., Park G. (2023). Effects of 20-hydroxyecdysone on UVB-induced photoaging in hairless mice. Biomed. Pharmacother..

[24] Lim H.S., Yoon K.N., Chung J.H., Lee Y.S., Lee D.H., Park G. (2021). Chronic Ultraviolet Irradiation to the Skin Dysregulates Adrenal Medulla and Dopamine Metabolism In Vivo. Antioxidants.

[25] Fang Y., Chen L., Wang X., Li X., Xiong W., Zhang X., Zhang Y., Han L., Cao K., Chen X. (2022). UVB irradiation differential regulate miRNAs expression in skin photoaging. An. Bras. Dermatol..

[26] Choi Y.J., Bae I.Y. (2023). White-spotted flower chafer (*Protaetia brevitarsis*) ameliorates inflammatory responses in LPS-stimulated RAW 264.7 macrophages. J. Insects Food Feed.

[27] Chin T., Lee X.E., Ng P.Y., Lee Y., Dreesen O. (2023). The role of cellular senescence in skin aging and age-related skin pathologies. Front. Physiol..

[28] Yang J.W., Fan G.B., Tan F., Kong H.M., Liu Q., Zou Y., Tan Y.M. (2023). The role and safety of UVA and UVB in UV-induced skin erythema. Front. Med..

[29] Hajialiasgary Najafabadi A., Soheilifar M.H., Masoudi-Khoram N. (2024). Exosomes in skin photoaging: Biological functions and therapeutic opportunity. Cell Commun. Signal..

[30] Bai G.L., Wang P., Huang X., Wang Z.Y., Cao D., Liu C., Liu Y.Y., Li R.L., Chen A.J. (2021). Rapamycin Protects Skin Fibroblasts From UVA-Induced Photoaging by Inhibition of p53 and Phosphorylated HSP27. Front. Cell Dev. Biol..

[31] Goldberg D.J., Amin S., Russell B.A., Phelps R., Kellett N., Reilly L.A. (2006). Combined 633-nm and 830-nm led treatment of photoaging skin. J. Drugs Dermatol..

[32] Glass G.E. (2021). Photobiomodulation: The Clinical Applications of Low-Level Light Therapy. Aesthet. Surg. J..

[33] Huang A., Nguyen J.K., Ho D., Jagdeo J. (2020). Light Emitting Diode Phototherapy for Skin Aging. J. Drugs Dermatol..

[34] Sorbellini E., Rucco M., Rinaldi F. (2018). Photodynamic and photobiological effects of light-emitting diode (LED) therapy in dermatological disease: An update. Lasers Med. Sci..

[35] Dunaway S., Odin R., Zhou L., Ji L., Zhang Y., Kadekaro A.L. (2018). Natural Antioxidants: Multiple Mechanisms to Protect Skin From Solar Radiation. Front. Pharmacol..

[36] Wei M., He X., Liu N., Deng H. (2024). Role of reactive oxygen species in ultraviolet-induced photodamage of the skin. Cell Div..

[37] Baumann L., Bernstein E.F., Weiss A.S., Bates D., Humphrey S., Silberberg M., Daniels R. (2021). Clinical Relevance of Elastin in the Structure and Function of Skin. Aesthet. Surg. J. Open Forum.

[38] Trębacz H., Barzycka A. (2023). Mechanical Properties and Functions of Elastin: An Overview. Biomolecules.

[39] Weihermann A.C., Lorencini M., Brohem C.A., de Carvalho C.M. (2017). Elastin structure and its involvement in skin photoageing. Int. J. Cosmet. Sci..

[40] Kim M.S., Chun K.E., Lee D.K., Song S.H. (2022). Evaluation of the Efficacy of an Elastin-Inducing Composition Containing Amino Acids, Copper, and Hyaluronic Acid: Results of an Open Single-Center Clinical Trial Study. Cosmetics.

[41] Imokawa G., Nakajima H., Ishida K. (2015). Biological mechanisms underlying the ultraviolet radiation-induced formation of skin wrinkling and sagging II: Over-expression of neprilysin plays an essential role. Int. J. Mol. Sci..

[42] Wang K., Meng X., Guo Z. (2021). Elastin Structure, Synthesis, Regulatory Mechanism and Relationship with Cardiovascular Diseases. Front. Cell Dev. Biol..

[43] Lee Y.I., Lee S.G., Ham S., Jung I., Suk J., Lee J.H. (2024). Exploring the Safety and Efficacy of Organic Light-Emitting Diode in Skin Rejuvenation and Wound Healing. Yonsei Med. J..

[44] Matsui T., Amagai M. (2015). Dissecting the formation, structure and barrier function of the stratum corneum. Int. Immunol..

[45] Champagne A.M., Muñoz-Garcia A., Shtayyeh T., Tieleman B.I., Hegemann A., Clement M.E., Williams J.B. (2012). Lipid composition of the stratum corneum and cutaneous water loss in birds along an aridity gradient. J. Exp. Biol..

[46] Woo Y.R., Kim H.S. (2024). Interaction between the microbiota and the skin barrier in aging skin: A comprehensive review. Front. Physiol..

[47] Sun W., He J., Zhang Y., He R., Zhang X. (2023). Comprehensive functional evaluation of a novel collagen for the skin protection in human fibroblasts and keratinocytes. Biosci. Biotechnol. Biochem..

[48] Shi J., Barakat M., Chen D., Chen L. (2018). Bicellular Tight Junctions and Wound Healing. Int. J. Mol. Sci..

[49] Ahn C., Shin D.H., Lee D., Kang S.M., Seok J.H., Kang H.Y., Jeung E.B. (2016). Expression of claudins, occludin, junction adhesion molecule A and zona occludens 1 in canine organs. Mol. Med. Rep..

[50] Chen T.C., Chang S.W. (2024). Non-lethal exposure to short-wavelength light-emitting diodes modulates tight-junction structure in human corneal epithelial cells via cAMP-dependent signaling. J. Photochem. Photobiol. B.

[51] de Paula-Silva M., Broering M.F., Scharf P., da Rocha G.H.O., Farsky S., Lino-Dos-Santos-Franco A. (2020). Red light-emitting diode treatment improves tissue recovery in DSS-induced colitis in mice. J. Photochem. Photobiol. B.

[52] Jomova K., Raptova R., Alomar S.Y., Alwasel S.H., Nepovimova E., Kuca K., Valko M. (2023). Reactive oxygen species, toxicity, oxidative stress, and antioxidants: Chronic diseases and aging. Arch. Toxicol..

[53] Milkovic L., Cipak Gasparovic A., Cindric M., Mouthuy P.A., Zarkovic N. (2019). Short Overview of ROS as Cell Function Regulators and Their Implications in Therapy Concepts. Cells.

[54] Zarkovic N. (2020). Roles and Functions of ROS and RNS in Cellular Physiology and Pathology. Cells.

[55] Pouremamali F., Pouremamali A., Dadashpour M., Soozangar N., Jeddi F. (2022). An update of Nrf2 activators and inhibitors in cancer prevention/promotion. Cell Commun. Signal..

[56] He F., Ru X., Wen T. (2020). NRF2, a Transcription Factor for Stress Response and Beyond. Int. J. Mol. Sci..

[57] Seol S.I., Kang I.S., Lee J.S., Lee J.K., Kim C. (2024). Taurine Chloramine-Mediated Nrf2 Activation and HO-1 Induction Confer Protective Effects in Astrocytes. Antioxidants.

[58] Saha S., Buttari B., Panieri E., Profumo E., Saso L. (2020). An Overview of Nrf2 Signaling Pathway and Its Role in Inflammation. Molecules.

[59] Galicia-Moreno M., Lucano-Landeros S., Monroy-Ramirez H.C., Silva-Gomez J., Gutierrez-Cuevas J., Santos A., Armendariz-Borunda J. (2020). Roles of Nrf2 in Liver Diseases: Molecular, Pharmacological, and Epigenetic Aspects. Antioxidants.

[60] Loboda A., Damulewicz M., Pyza E., Jozkowicz A., Dulak J. (2016). Role of Nrf2/HO-1 system in development, oxidative stress response and diseases: An evolutionarily conserved mechanism. Cell. Mol. Life Sci..

[61] Shin D., Lee S., Huang Y.H., Lim H.W., Lee Y., Jang K., Cho Y., Park S.J., Kim D.D., Lim C.J. (2018). Protective properties of geniposide against UV-B-induced photooxidative stress in human dermal fibroblasts. Pharm. Biol..

[62] Yi R., Zhang J., Sun P., Qian Y., Zhao X. (2019). Protective Effects of Kuding Tea (Ilex kudingcha C. J. Tseng) Polyphenols on UVB-Induced Skin Aging in SKH1 Hairless Mice. Molecules.

[63] Orhan C., Gencoglu H., Tuzcu M., Sahin N., Ozercan I.H., Morde A.A., Padigaru M., Sahin K. (2021). Allyl isothiocyanate attenuates LED light-induced retinal damage in rats: Exploration for the potential molecular mechanisms. Cutan. Ocul. Toxicol..

[64] Wang C.Y., Tsai S.C., Yu M.C., Lin Y.F., Chen C.C., Chang P.C. (2015). Light-emitting diode irradiation promotes donor site wound healing of the free gingival graft. J. Periodontol..

[65] Salman S., Guermonprez C., Peno-Mazzarino L., Lati E., Rousseaud A., Declercq L., Kerdine-Römer S. (2023). Photobiomodulation Controls Keratinocytes Inflammatory Response through Nrf2 and Reduces Langerhans Cells Activation. Antioxidants.

